# Psoas Abscess From Spinal Tuberculosis Mimicking Appendicitis: A Case Report

**DOI:** 10.7759/cureus.97485

**Published:** 2025-11-22

**Authors:** Abdalla Fathi Salih, Shameen Malik

**Affiliations:** 1 Internal Medicine, East Lancashire Hospitals NHS Trust, Blackburn, GBR

**Keywords:** a case report, computed tomography guided aspiration, isoniazid resistant, multidisciplinary decision-making, right lower quadrant abdominal pain, secondary psoas abscess, spinal tuberculosis

## Abstract

Osteoarticular tuberculosis (TB) is a form of extrapulmonary TB that affects bones and joints, with the spine being the most common site of involvement. Spinal TB, also known as Pott’s disease, is therefore the most frequent manifestation of osteoarticular TB. Historically, Pott’s disease has been more prevalent in developing countries; however, its incidence is increasing in developed nations, driven by global migration and a growing population of immunocompromised individuals.

We report the case of a woman in her 50s who presented with a one- to two-month history of intermittent lower abdominal pain, radiating to the back and associated with fever, nausea, and recent weight loss. Initial examination suggested acute appendicitis, with tenderness localised to the right lower quadrant and McBurney’s point. Laboratory investigations revealed mild anaemia (haemoglobin, or Hb 95 g/L) and elevated C-reactive protein (CRP) (49 mg/L), while other parameters were normal. Computed tomography (CT) imaging revealed a large left psoas collection with vertebral destruction at L4, suggestive of discitis/osteomyelitis. Magnetic resonance imaging (MRI) confirmed a multiloculated left psoas abscess with extensive involvement of the L4/L5 vertebrae, paravertebral tissues, and epidural space.

CT-guided aspiration obtained approximately 90 mL of pus, which tested positive for *Mycobacterium tuberculosis* without rifampicin resistance. Initial empiric therapy with flucloxacillin was discontinued, and antitubercular therapy was commenced. Subsequent drug susceptibility testing revealed isoniazid resistance, prompting regimen modification to include rifampicin, pyrazinamide, ethambutol, and levofloxacin. The patient improved clinically, with gradual resolution of symptoms and reduction in abscess size on follow-up imaging. Multidisciplinary management, involving general surgery, radiology, microbiology, and infectious disease teams, was critical for timely diagnosis and tailored therapy.

This case highlights the atypical presentation of spinal TB with a left-sided psoas abscess mimicking acute appendicitis. It also shows the importance of a multidisciplinary approach, early imaging, CT-guided aspiration for microbiological confirmation, and culture-guided therapy, especially in the context of drug-resistant TB. Awareness of such presentations can prevent diagnostic delays, reduce morbidity, and optimise outcomes.

## Introduction

Osteoarticular tuberculosis (TB) is a form of extrapulmonary TB that affects bones and joints, with the spine being the most common site of involvement (50%-60% of cases) [1,2]. Spinal TB, also known as Pott’s disease, is therefore the most frequent manifestation of osteoarticular TB. Historically, Pott's disease has been more prevalent in developing countries; however, its incidence is increasing in developed nations, driven by global migration and a growing population of immunocompromised individuals [3]. As of 2023, spinal TB accounted for 4.4% of cases in the UK [4]. It was first described by Percival Pott in 1779 and accounts for approximately 1%-2% of all TB cases globally [5-7].

Psoas abscess, first described by Mynter in 1881 as “psoitis,” is a complication of spinal TB [8,9]. It involves the accumulation of pus within the psoas muscle compartment and is often overlooked due to its nonspecific clinical features [10]. The “classic triad” of fever, back (or flank) pain, and limp is seen in 30% of cases [11].

In this report, we present a case of spinal TB complicated by a psoas abscess, which presented in an unusual manner and posed diagnostic challenges. This case also highlights important therapeutic considerations in the management of such conditions.

## Case presentation

A lady in her 50s presented to the Emergency Department with a one- to two-month history of intermittent lower abdominal pain. She described the pain as severe and radiating to the back. She stated that the pain is aggravated by eating and movement and is associated with fever and nausea; however, she reported that there have been no episodes of vomiting. She also reports recent weight loss. She reported no shortness of breath, chest pain, or hematemesis. She stated that she had a recent travel history to Pakistan; however, she denies being in contact with anyone ill during this time. The patient has a past medical history of gallstones, gastritis, and type 2 diabetes mellitus.

On examination, the patient's vitals were stable, with a heart rate of 90 beats per minute, blood pressure of 118/75, oxygen saturations of 97%, and a Glasgow Coma Scale of 15/15. Abdominal examination showed tenderness along the lower abdomen, mainly in the right lower quadrant, and tenderness at McBurney’s point. Blood results showed haemoglobin (Hb) 95 (normal range: 115-165 g/L), white blood cell (WBC) 6.3 (normal range: 4.0-11.0 × 10^9^/L), C-reactive protein (CRP) 49 (normal range: <3.3 mg/L), amylase 36 (normal range: 30-118 IU/L), and normal liver and renal function tests (Table 1).

**Table 1:** Blood results on admission day ALT, Alanine Aminotransferase; CRP, C-Reactive Protein; eGFR, Estimated Glomerular Filtration Rate; Hb, Haemoglobin; MCV, Mean Corpuscular Volume; MCH, Mean Corpuscular Haemoglobin; MCHC, Mean Corpuscular Haemoglobin Concentration; TB, Tuberculosis; WBC, White Blood Cell

Blood Test Parameters	Result	Reference Value
Full Blood Count		
Hb	95	115 - 165 g/L
WBC	6.3	4.0 - 11.0 × 10^9^/L
Platelets	239	150 - 450 × 10^9^/L
MCV	71.4	76.0 - 100.0 fL
MCH	22	27.0 - 32.0 pg
MCHC	308	310.0 - 360.0 g/L
Neutrophils	4.3	2.0 - 7.5 × 10^9^/L
Lymphocytes	1.4	1.5 - 4.0 × 10^9^/L
Renal Function Test		
Sodium	140	133 - 146 mmol/L
Potassium	4.1	3.5 - 5.3 mmol/L
Urea	4.4	2.5 - 7.8 mmol/L
Creatinine	51	49 - 90 mmol/L
eGFR	>90	>90 mL/min/1.73 m^2^
Liver Function Test		
Total bilirubin	5	0 - 20 umol/L
ALT	13	7 - 40 IU/L
Alkaline phosphatase	11	30 - 130 IU/L
Albumin	38	35 - 50 g/L
CRP	49	<3.3 mg/L
Amylase	36	30 - 118 IU/L

**Figure 1:**
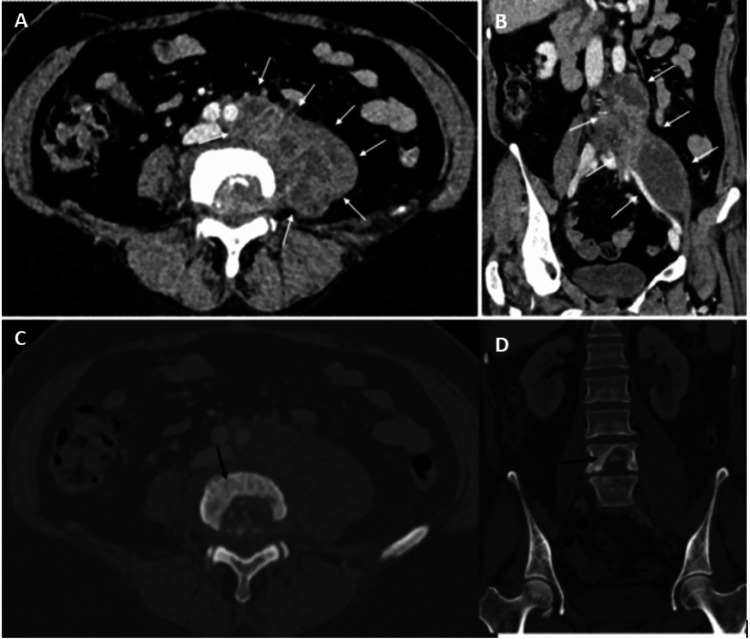
Axial and coronal images Axial (A) and coronal (B) images from a portal venous phase CT scan show a large left psoas abscess (white arrows) and a destructive lesion in the L4 vertebral body (black arrows), which is better appreciated on the bone-window axial (C) and coronal (D) images. CT, Computed Tomography

Initially, appendicitis was suspected, and a computed tomography (CT) scan was requested, which showed a large left psoas collection with a destructive process involving the L4 vertebra, highly suggestive of discitis/osteomyelitis (Figure 1). An urgent magnetic resonance imaging (MRI) scan and a discussion with neurosurgery were recommended by the Radiology Department.

**Figure 2:**
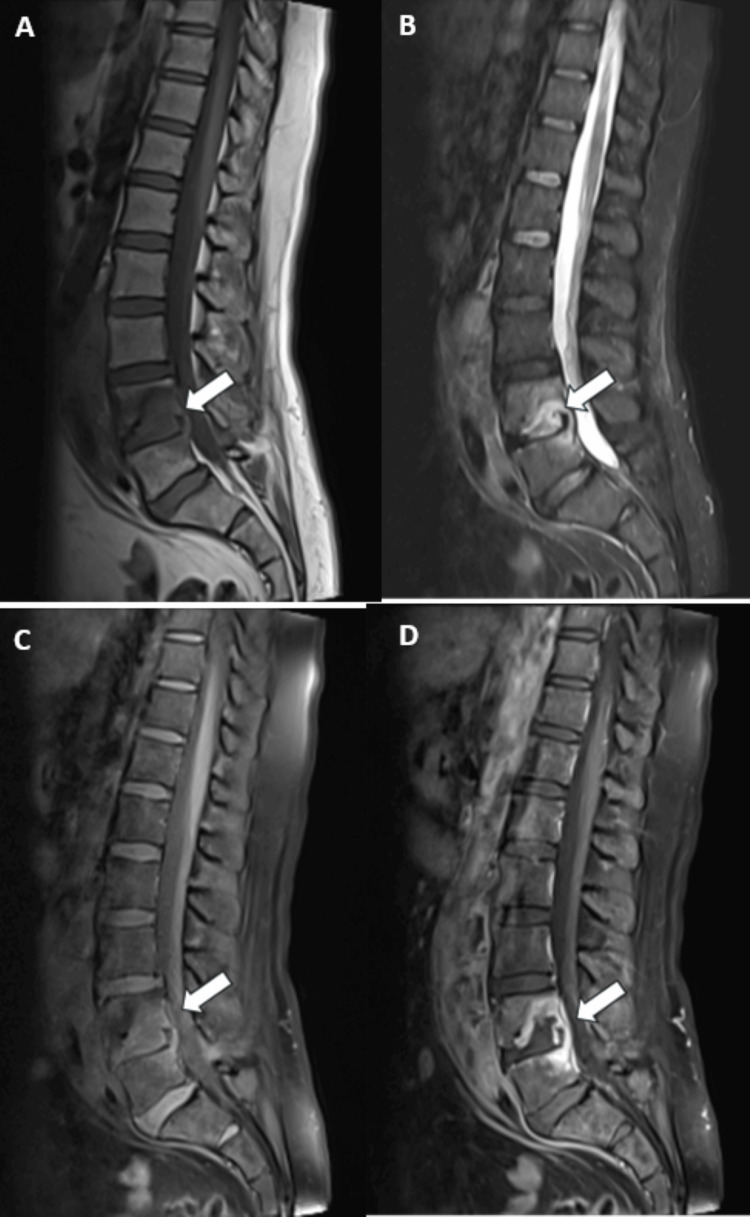
From left to right (A-D): sagittal T1, STIR, fat-suppressed T1 pre-contrast, and fat-suppressed T1 post-contrast images Destructive changes are seen in the posterior vertebral body and inferior endplate of the L4 vertebra, with a relatively preserved L4/L5 intervertebral disc (A-D). Oedematous changes - low signal on T1 (A) and high signal on STIR (B) - are present in the surrounding bone marrow of the L4 vertebral body, as well as along the posterior superior endplate of the L5 vertebra. The bony abnormality is associated with a multiloculated collection. The bony abnormality, as well as the psoas collection, shows heterogeneous peripheral enhancement on post-contrast fat-suppressed T1-weighted images. STIR, Short Tau Inversion Recovery

After consulting with Neurosurgery, it was agreed that the patient would be admitted under General Surgery while awaiting the MRI to investigate the destructive lesion further. She was started on flucloxacillin as empiric treatment for discitis. MRI results showed a large multiloculated left psoas abscess, with additional extensive infective/abscess disease involving the L4/L5 disc space, the body of L4, the paravertebral soft tissues, and intraspinal disease - particularly involving the anterior epidural space, retroperitoneal nodes, the posterior body of L5, and the left upper sacrum - which was highly suggestive of TB (Figure 2).

**Figure 3:**
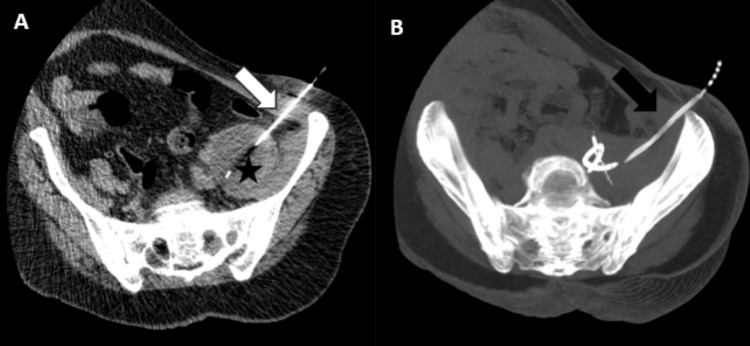
CT-guided catheter insertion in the left psoas abscess Axial (A) and axial maximum intensity projection (MIP) (B) images show a needle (white arrow), followed by a catheter (black arrow), within the left psoas abscess (*). CT, Computed Tomography

The patient was then tested for human immunodeficiency virus (HIV) and co-existing pulmonary TB according to national guidelines, with a chest X-ray being performed, which showed no findings of TB; three acid-fast bacilli (AFB) sputum tests, which came back negative; and an HIV test, which also came back negative. Initially, the abscess was deemed undrainable due to its location, as a fine needle would not aspirate an adequate amount. A Multi-Disciplinary Team meeting was held involving the specialties directly involved in the patient’s care (General Surgery, Radiology, Microbiology, and the TB team), which highlighted the importance of obtaining a sample in order to start the patient on anti-TB medication. Ultimately, it was agreed that a sample could be obtained using CT-guided catheter insertion. A CT-guided aspiration was performed, and approximately 90 mL of thick, odourless pus was aspirated to dryness and sent for MC&S, TB culture, and cytology, and a drain was left in place (Figure 3).

The sample that was aspirated tested positive for *Mycobacterium tuberculosis*, and rifampicin resistance was not detected. The sample was then sent to the regional reference lab, where TB whole genome sequencing is performed.

After the aspiration was completed, the patient was started on TB medication, which consisted of a fixed-dose combination pill containing rifampicin, ethambutol, isoniazid, and pyrazinamide, and she was also started on pyridoxine to prevent neuropathy and neurotoxicity associated with TB medication, as previously advised by the TB team. The patient was deemed medically optimised for discharge, and appropriate follow-up was arranged for her to be seen in two weeks in the TB clinic. Follow-up was also arranged with the General Surgical Department, as the patient was being discharged with a drain, which would be cared for with the help of district nurses, and adequate review after discharge was required to determine when the drain could be removed.

**Figure 4:**
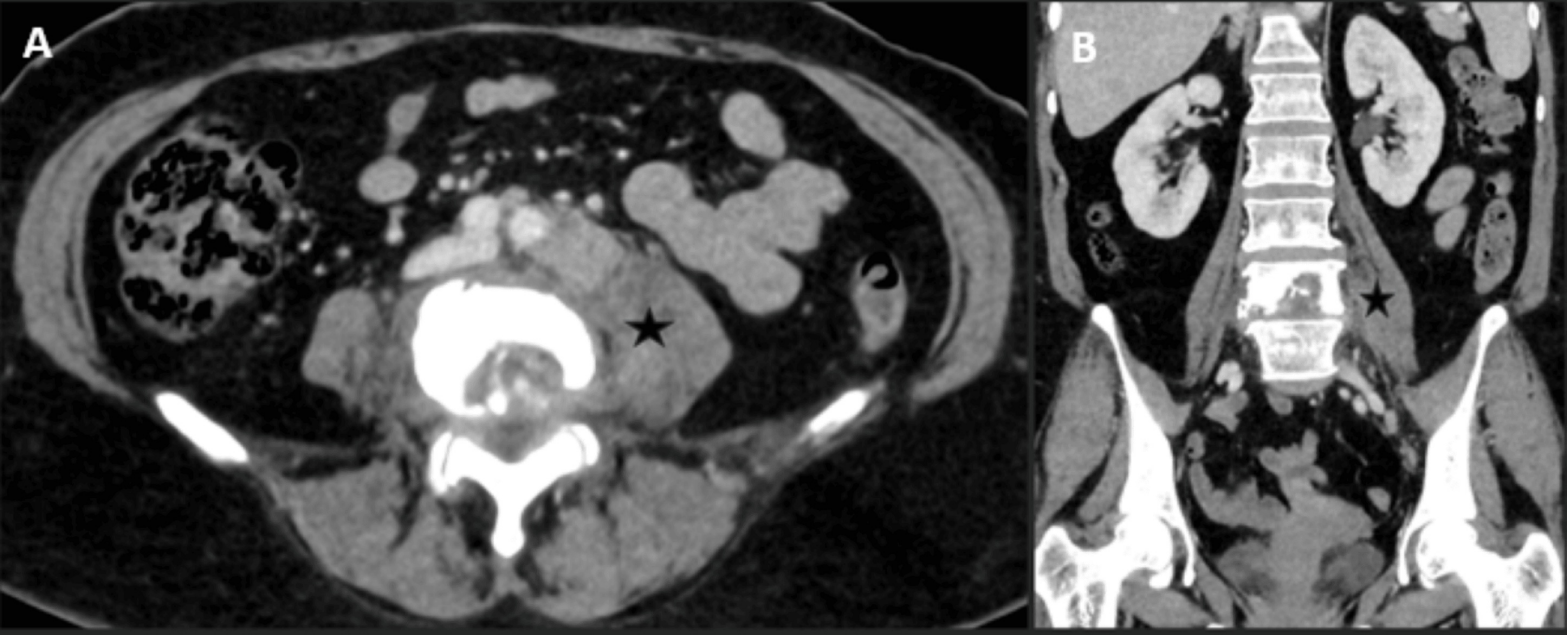
Axial and coronal images Axial (A) and coronal (B) images from a follow-up CT scan show a marked interval reduction in the size of the left psoas abscess (*). CT, Computed Tomography

The patient stated in her initial follow-up that her physical health had improved compared to when her symptoms first started. She stated that the pain was improving and that, due to this, she could mobilise. She also reported that she was not losing weight as mentioned previously; however, her appetite had remained the same. Culture results showed isoniazid resistance with a fabG1 mutation, and her medications were adjusted accordingly, placing her on rifampicin, pyrazinamide, ethambutol, and levofloxacin for a total duration of six months, with regular follow-up in the clinic every six to eight weeks. Initial review by general surgeons in the clinic showed that the drain output at that time was around 20 mL of cream-coloured fluid. Physical examination showed that the abdomen was soft and non-tender, with no abnormalities detected. A follow-up CT was arranged two months after the patient initially presented to the Emergency Department to review the abscess. It showed a reduction in the size of the abscess; however, the bone destruction at L4 and the posterior body of L5 remained unchanged, and additional TB changes were noted in the left side of S2 (Figure 4).

A second review was arranged by the general surgeons, and at that stage, the patient’s drain output was minimal, at around 5-10 mL, and the patient denied any fever, nausea, vomiting, or abdominal pain. It was deemed that the drain could be removed, and the patient was discharged from the general surgeons' care.

## Discussion

Psoas abscess can be classified into primary or secondary infection. Primary psoas abscess is usually due to haematogenous spread from a distant source, and risk factors include renal failure, diabetes mellitus, HIV, IV drug use, and malignancy [11,12]. Primary psoas abscesses are typically monomicrobial, often caused by *Staphylococcus aureus*. A multiple-case series reported that *S. aureus* was responsible for approximately 42% of primary psoas abscess cases [13].

Secondary psoas abscess results from the contiguous spread of infection from adjacent structures, such as the spine, gastrointestinal tract, or genitourinary system. Secondary abscesses are far more common globally, with spinal TB (Pott’s disease) being a leading cause - particularly in developing countries - where approximately 5% of Pott’s disease cases develop a psoas abscess [14]. In contrast, in case reports from developed settings, Crohn’s disease has been reported as the most frequent underlying cause [10,15,16]. Other risk factors include previous surgical procedures or instrumentation in the groin, lumbar, or hip regions [12].

Secondary psoas abscesses are often polymicrobial, with *Escherichia coli* commonly isolated from gastrointestinal or urinary sources, whereas *S. aureus* predominates in abscesses of skeletal origin, such as vertebral osteomyelitis. Other pathogens reported include *Bacteroides* species and *M. tuberculosis*, particularly in TB-endemic regions [10,13]. The polymicrobial nature of secondary abscesses highlights the importance of culture-guided therapy.

Delayed diagnosis of Pott's disease leads to neurological complications, including spinal cord compression [17]. Psoas abscess can lead to septic shock (20% of cases), pelvic venous thrombosis (due to compression of veins), arthritis, and paralytic ileus [18,19].

In this case, the patient presented in an atypical manner, with initial suspicion of acute appendicitis due to right lower quadrant tenderness and fever. Another differential, after the CT finding of a left psoas abscess, was a pyogenic psoas abscess caused by *S. aureus*; therefore, IV flucloxacillin was started for the patient as empirical treatment.

Psoas abscess has been reported to mimic appendicitis in previous cases. For example, Vasigh et al. described an isolated psoas abscess caused by *M. tuberculosis*, and Arroyo-Rangel et al. reported a primary psoas abscess caused by *S. aureus*, both presenting with right lower quadrant pain and systemic features suggestive of appendicitis. Unlike our case, both abscesses were primary and showed no vertebral involvement [20,21].

Byamukama et al. described a case of Pott’s disease with a large paravertebral abscess, presenting with right lower quadrant pain, similar to our patient. However, the patient presented in a resource-limited rural setting in Uganda and had AFB confirmed within the sample aspirate. While both cases showed advanced disease with contiguous spread beyond the vertebral bodies, the pattern of extension differed, and the patients had different clinical consequences. The absence of neurological symptoms in our patient, despite significant vertebral and epidural extension, underscores the heterogeneous clinical expression of Pott’s disease and the critical role of early imaging in identifying subclinical spinal involvement before irreversible neurological damage occurs [22].

A case report by Miyakoda et al. was similar to our case, as it highlighted the diagnostic challenges of extrapulmonary TB, particularly when pulmonary involvement and sputum AFB positivity are absent. In both patients, CT-guided aspiration of the psoas abscess was the key diagnostic step, confirming TB. However, in Miyakoda et al.’s case, infection at the L3/L4 vertebral level was complicated by central nervous system dissemination, presenting as TB meningitis, ultimately resulting in a poor outcome despite treatment. Our case showed that early multidisciplinary involvement, timely imaging, and microbiological confirmation via CT-guided aspiration can improve prognosis [23].

A case published by Meena et al. also demonstrated that abdominal pain can be the primary presenting feature of spinal TB, often diverting clinicians toward gastrointestinal or visceral causes and delaying diagnosis. Their case relied on MRI findings and clinical suspicion to initiate TB treatment, whereas our case highlights the benefit of direct sampling via CT-guided aspiration, which allows microbiological confirmation, drug sensitivity testing, and more precise therapy, especially when pulmonary TB tests are negative. In our patient, isoniazid resistance was detected, and, therefore, it was switched to levofloxacin [24]. This highlights the importance of culture-guided management.

It is important to test for drug sensitivities, as there is an increase in TB strains resistant to isoniazid. It is estimated that around 8% of TB patients worldwide have isoniazid-resistant TB (Hr-TB) [25]. As per WHO guidelines, the standard regimen for Hr-TB is six months of RZELfx, which was started in our patient. There is debate about the duration of treatment when there is bone involvement, particularly in spinal TB. The recommended duration across different guidelines ranges widely, from as short as six months up to 18 months or more [7]. 

In the Elgendy et al. case, the patient presented with abdominal pain and bilateral flank swelling as the initial symptoms, corresponding to large bilateral psoas abscesses that were easily drained percutaneously under ultrasound guidance. In contrast, our patient had a deep-seated, multiloculated abscess, initially deemed non-drainable, requiring CT-guided aspiration after MDT discussion [26].

## Conclusions

In summary, this case demonstrates that spinal TB complicated by a psoas abscess can present atypically, with abdominal pain as the predominant symptom, mimicking appendicitis even when the abscess is left-sided. CT-guided aspiration should be strongly considered, as it enables definitive microbiological diagnosis, drug sensitivity testing, and targeted management. The identification of isoniazid resistance in our patient emphasises the growing importance of culture-guided management in TB cases. Early multidisciplinary collaboration among radiology, surgery, infectious diseases, and microbiology teams was crucial in optimising outcomes and preventing long-term complications or disability.
